# The Institutional Worker

**Published:** 1912-12-28

**Authors:** 


THE
### INSTITUTIONAL WORKER
Being a Special Supplement to "The Hospital"

### BUREAU OF INFORMATION.

### Rules for Correspondents.

1. Every letter, on whatever subject, must be accompanied by the coupon to be cut from the back cover (inside page) of The Hospital, current issue, and must contain the name and address of the correspondent with pseudonym for publication if desired.

2. Replies by post cannot be given, save under exceptional circumstances at the Editor's discretion.

3. Letters from Approved Homes in reply to special needs published in the Bureau should state terms and full particulars, and be sent prepaid under cover to the Editor of the Bureau with name written across coupon for identification.

4. Proprietors of Homes which have not yet been entered on the List of Approved Homes, but have spare accommodation likely to suit special needs, are invited to write for an application form for registration. The fee for registration, which includes two announcements of the Home in the Bureau, is 5s.

5. Special needs of every description relating to those who are sick or in difficulties are made known in these columns free of charge.

6. All communications to be addressed to The Editor of The Hospital, 28 Southampton Street, Strand, London, W.C., and marked "Bureau of Information."

### Approved Homes.

Note.—*No Home is added to the List without direct medical and other testimony to its good management.*

**Dover.**—7 Waterloo Crescent. Principals: The Misses Elliott. Medical, surgical, rest cure, maternity cases. Excellent accommodation for permanent patient in good room overlooking sea, with south aspect.

### Hospital Economics.

**Cost of Sanatoriums for Children.**—Your query is very interesting in view of recent developments. There are at present two sanatoriums devoted exclusively to the treatment of tuberculous children—viz. the Children's Sanatorium, Holt, Norfolk, with twenty beds, and the Harpenden Sanatorium, in connection with the National Children's Home and Orphanage. The expenditure on the latter, which offers accommodation to fifty patients, besides officers, was close on £10,000, and this coincides with the estimate of about £200 a bed for a permanent building furnished by another competent authority. You could deduct the cost of site from this estimate, as we understand from you that would be presented, and for an institution with 100 beds the cost would be proportionally less. As regards maintenance, in the report for the year 1911 of the Holt Sanatorium the average cost of each patient per week is stated to be £1 6s. 6d.; these figures are, however, not conclusive, as they are arrived at under circumstances of a temporary nature. The average cost at Harpenden is not yet ascertainable, as the new building has only just been taken over. Very good results have been obtained in temporary buildings and in houses adapted for the purpose, and the Honorary Secretaries of either of the above institutions would give you further particulars on this point.—H. B.

**Administration of Cottage Hospital.**—You are paying far too high. The cost of management is usually reduced to an almost negligible amount by honorary service in the case of hospitals containing less than twenty beds. Clerical assistance to the extent of £20 or £30, with a small outlay on postages and stationery, should suffice. The matron is the proper person to undertake the small amount of official correspondence, and on the occasion of any special appeal or festival, help should be given her.—Subscriber.

### Needs and Helpers.

**Change of Air.**—The patient is a young man who has exhausted his savings during the last twelve months in medical treatment for dilation of the stomach. He is recommended change of air, but has no friends or relatives who can help him. Write to the Hon. Founder, St. John's Home, Beach Road, Southsea, and give full particulars, including age and previous occupation.—H. J., Leeds.

**Inexpensive Sanatorium Treatment.**—Write to Dr. Esther Carling, Maitland Sanatorium, Peppard Common, Oxon. The terms are 30s. a week for adults, but separate accommodation for ladies is from £3 3s. to £5 5s. There is nothing lower than this. Would your sister be happy among working women? If you cannot pay for a private room she would, in our judgment, do better in one of the Nursing Homes we have mentioned, where even with doctor's fees in addition there might still be a balance on the right side, as compared with the necessarily costly sanatorium treatment. Have you thought of applying to the Queen Alexandra Sanatorium at Davos Platz, Switzerland? The terms there are £2 2s. a week.—J. M. S. (140a).

**Address of Home for Incurables.**—Can any of our readers inform us whether the "Turner Memorial Home" near Liverpool is still in existence? We are making inquiries for you, and will let you know the result.—Miss V.

**Inexpensive Home for Mild Mental Case.**—The patient is forty years of age, and has hitherto earned her living as useful companion. She has no means, and is dependent on her sister, who is herself in a situation. It is desired to place her in the house of a nurse for small payment, where she could give her services in household matters, of which she is quite capable. Can anyone help?—Stella (141x).

### Training and Employment.

**Ling's Swedish System.**—There are several good colleges in this country for learning gymnastics under this system, but possibly the Anstey Physical Training College, Chester Road, Erdington, may suit you best. The course of training extends over two years. The qualification to aim at is the "Ling Diploma," which enables you to become a member of the Ling Association. Massage and medical gymnastics can be taken as part of the course.—Elsie P., Birmingham.

**Lady Inspectors of Factories and Workshops.**—We fear you will have some difficulty in obtaining this position. There are only eighteen lady inspectors altogether (eleven junior, six senior, and one principal), so that vacancies rarely occur. When they do occur they are filled by competitive examination among candidates nominated by the Home Secretary. The prescribed age for candidates is between twenty-five and forty. If you wish to try you can obtain an Application Form for Nomination from the Home Office.—P. N.

### Miscellaneous.

**Address of Valuer for Nursing Home.**—Address wanted of reliable firm to value goodwill and furniture of Nursing Home for prospective purchaser. We are taking steps to procure this information for you, and under the circumstances of urgency will communicate direct if you will furnish stamped envelope for reply.—K. P.

### Nursing in the Empire and Abroad.

**Climate in Jamaica.**—There is comparatively little malaria, and your friends would run no risks, even during the hot months, June to September. Typhoid is still very prevalent, and trained nurses are in request for this. But the supply is fairly good, and you could not count on regular employment during your stay in the island. In winter and early spring the climate is one of the finest in the world.—MADELINE.

### Letter-Box.

**The Editor acknowledges communications with enclosures from:**— J. B., Maidstone; L. S. Z., Brockley; the Rev. J. Pullein-Thompson.

**Communications have been received and attended to from:**—Miss T., Hove; Miss E. J. S., Blackheath; T. H. W., Norfolk; Sister K., Falmouth; the Misses I. and S., Lytham ; Mrs. S., Musselburgh; Miss W. P., Udimore.

**The Editor is much indebted for testimonials received in response to inquiries from:**—Rev. H. G Daniell; Dr. Hill Joseph; Dr. Murphy; Dr. Boothroyd; Miss Darling.

**Persons requiring nurses and nurses requiring appointments will find their needs met in the adjoining pages, where many useful announcements appear this week.**

### Housekeeping Queries.

**Fruit Salads.**—To be really nice this should be made some hours before it is required, and the fruit should be arranged in layers with sugar between, and should be stirred very little until just before dishing; the flavour is much more delicate if made in this way, and is better blended. Grapes should always be skinned before using for salad. Every kind of fruit which can be eaten raw is useful, and a few nuts may be added.—HOME SISTER.

**Variety in Puddings.**—Will the following recipes help you? They are quite inexpensive and not troublesome. [Others will appear next week.—ED.]

**Bread Caramel.**—5 oz. stale breadcrumbs (or 3 oz. breadcrumbs, 2 oz. stale sponge-cake crumbs), 3 oz. loaf sugar, 2 tablespoonfuls of water, 2 eggs, 1 tablespoonful castor sugar, ½ pint milk, 1 teaspoonful vanilla essence. Put the bread (and cake) crumbs into a bowl, the lump sugar and water into a saucepan, shake it over the fire to melt and become a dark brown. When this is a nice coffee colour pour it into a basin on the milk; it will become hard, but after a few minutes this will dissolve again. Pour it on the breadcrumbs, stir well, add castor sugar, two yolks of eggs, essence; let this soak for half-hour, then beat up the whites very stiffly, fold carefully into the mixture, put into a fancy mould that has been thickly smeared with butter and then well cover with castor sugar; cover with buttered paper and steam—not boil—very gently for one hour; turn out carefully, as it should be very light. Serve, if liked, with custard sauce.

### Institution Facts and Figures.

### AN INVITATION TO THE PRACTICAL WORKER.

IN every well-managed institution there is an accumulation of valuable observations, extending often over a term of years, which are wasted because there is no medium through which they can be brought to the notice of fellow-workers in the same field. It is our hope through this section of the "Institutional Worker" to collect some of these facts and present them to our readers. Every month two questions will be set relating to the internal working of institutional life, its cost, and distinctive features. The best answers will be published in due course, and payment will be made for contributions at the rate of Five Shillings for each answer which the Editor considers of sufficient interest for publication.

QUESTIONS FOR DECEMBER.

1. What was the exact amount of milk consumed in a given week in December in your institution (*a*) in a medical ward; (*b*) in a surgical ward? State daily average of patients. Describe the system in use for checking the amount used. What was the average cost per head of each patient in those wards respectively for milk during that week calculated on the contract price?

2. What is the most popular supper dish (savoury) in your institution? Give the recipe and quantity of each ingredient required for fifty persons, and state the cost per head.

The figures published by King Edward's Hospital Fund relating to the cost of various articles in London hospitals have brought forcibly before the public the variations which occur in the cost of everyday articles in London institutions. If our readers will help us we hope to throw additional light on the hospital commissariat throughout the kingdom.

Answers are invited from workers in every class of institution for the sick, and the value of those published will be greatly enhanced if opportunity be afforded for contrasting figures in various parts of the kingdom. The name of the institution need not be mentioned unless desired. The only condition is that the figures must be the result of actual observation and computation, not estimates. Either or both of the questions may be answered by each contributor, and payment will be made for all answers published as above mentioned.

The following rules must be observed by contributors to the FACTS AND FIGURES Column:—

1. Answers must be written on one side of the paper and must bear the name and address of the sender and be accompanied by coupon to be cut from the back cover (inside page) of the current issue of THE HOSPITAL. A pseudonym must be chosen if the name is not to be published.

2. Answers must be addressed to the Editor of THE HOSPITAL, 28-29 Southampton Street, Strand, London, W.C.; they must be marked in the left-hand corner "Facts and Figures," and must be received before January 7.

### Editor's Notices.

**Contributions:**

Contributions should be written, or preferably typed, on one side of the paper only, and all articles sent in are accepted upon the distinct understanding that they are forwarded to THE HOSPITAL only.

The Editor cannot undertake to return MSS. not used, but when a stamped directed envelope is enclosed the MSS. may be returned if a special request is made.

Accepted articles and paragraphs of news will be paid for after publication at the scale rate.

**Address:**

To prevent delay all contributions and letters on editorial business must be addressed exclusively to the Editor, "The Hospital," 29 Southampton Street, Strand, London, W.C. It is important that this regulation shall be strictly observed.

**Correspondence:**

Correspondence on all subjects is invited. The name and address of correspondents must be given as a guarantee of good faith, but not necessarily for publication.

**Special Articles:**

Special articles are invited, and questions, inquiries, and paragraphs upon all matters relating to the work, administration, and management of general, special, mental, fever, cottage and convalescent hospitals, sanatoria, homes, institutions, societies and organisations for the treatment or care of the sick, injured and dependants of all classes. Every matter affecting the interests and welfare of the staff of all grades working in these institutions will receive special consideration.

**Administrative Medicine, Research and Items of News:**

Special terms will be paid for approved articles on subjects in this wide field by experts who have made some section of it their special study and interest. We wish to give prominence to every movement tending to advance accuracy of diagnosis, efficiency in treatment, and the development of modern methods to eradicate disease and promote the welfare of the suffering.

**A Bureau of Information:**

We invite inquiries and applications for information and help in providing for that numerous class of sufferers who require special care or house-room which they cannot obtain in their own homes or secure for themselves. **We cannot, however, prescribe, or recommend practitioners.**

**Books for Review:**

Publishers are particularly requested to send advance proofs of any new books of importance whenever possible, as the Editor has made arrangements to publish immediate reviews on a new plan.

**Photographs, Plans, Blocks, and Illustrations:**

It is requested that wherever possible MSS. may be accompanied by illustrations in any of the above forms. The name of the sender and of the article to which it belongs should be written on the back of each photograph, drawing, or block for purposes of identification.

**Local Papers:**

Newspapers containing reports or news paragraphs should be marked and addressed to the Sub-Editor.

**The Coupon System:**

A coupon will be found at the bottom of the third inside page of the cover each week. This coupon must be attached to every question or inquiry to which an answer is desired in THE HOSPITAL.

### Be Prepared.

THE circulation of THE HOSPITAL is so rapidly increasing that it is often a matter of considerable difficulty to secure a copy of the Journal at the local newsagents, especially in some of the smaller provincial towns. In these circumstances, we would strongly recommend our readers to *be prepared* against the contingency of THE HOSPITAL being sold out by subscribing either through a bookseller or direct to the Publishing Offices, by which means you are sure to obtain a copy of the Journal almost immediately upon publication. It reaches you regularly on a certain day and at a fixed hour; you know when you may expect it, and there is no fear of being told that "it is out of print."

This is a great help when seeking a post, as it enables you to send in your application without delay, an advantage which may secure you the appointment. In addition to this, as a Subscriber, no matter how many times you may change your address, or in what part of the country you may be at the moment, a postcard will ensure that you receive THE HOSPITAL on the same day and at the same hour as when at home.

**Subscriptions,** which may commence at any time, are payable in advance. Cheques and Post-Office Orders should be crossed London County and Westminster Bank, Covent Garden Branch, and made payable to the Manager of THE HOSPITAL.

RATES OF SUBSCRIPTION (payable in advance).

| | | |
|---|---|---|
| **UNITED KINGDOM.** | | |
| Three Months (including Postage) | ... ... ... ... | 2s. 0d. |
| Six Months do. | ... ... ... ... | 4s. 0d. |
| Twelve Months do. | ... ... ... ... | 6s. 6d. |
| **FOREIGN AND COLONIAL.** | | |
| Three Months (including Postage) | ... ... ... ... | 2s. 6d. |
| Six Months do. | ... ... ... ... | 5s. 0d. |
| Twelve Months do. | ... ... ... ... | 8s. 8d. |

*SUBSCRIPTION FORM.*

*Please send me* THE HOSPITAL *for..............months, commencing with your issue of.............................., for which please find enclosed............................*

*Name*..................................................

*Address* ..................................................

..................................................

*Date*..................................

*To* .................................. *Newsagent.*

### Manager's Notices.

**All letters on business matters must be addressed to The Manager, "The Hospital," 28 and 29 Southampton Street, London, W.C.**

**Advertisements:**

To ensure insertion, all advertisements for THE HOSPITAL must reach the Manager not later than Wednesday at noon in each week. For scale of charges see pages v and 4.

**Remittances:**

**It is especially requested that remittances be made by Cheque or Postal Order, and in halfpenny stamps only when the amount is under sixpence.**

